# Supplementary material for: Fabric-based lamina emergent MXene-based electrode for electrophysiological monitoring
Source: Nat Commun. 2024 Oct 2;15:5974. doi: 10.1038/s41467-024-49939-x (PMC11446925; doi:10.1038/s41467-024-49939-x)
Supplement: Supplementary file 1 — Supplementary Information [file 41467_2024_49939_MOESM1_ESM.pdf]

Supplementary Information

Fabric-Based Lamina Emergent MXene-Based Electrode for Electrophysiological Monitoring

Sanghyun Lee,[1,†] Dong Hae Ho,[2,†] Janghwan Jekal,[3,†] Soo Young Cho,[1] Young Jin Choi,[1] Saehyuck Oh,[3] Yoon Young Choi,[4] Taeyoon Lee,[5,6] Kyung-In Jang,[3,*] and Jeong Ho Cho[1,*]

[1]Department of Chemical and Biomolecular Engineering, Yonsei University, Seoul 03722, Republic of Korea
[2]Department of Energy Science and Engineering, Daegu Gyeongbuk Institute of Science and Technology (DGIST), Daegu, 42988, Republic of Korea
[3]Department of Robotics and Mechatronics Engineering, Daegu Gyeongbuk Institute of Science and Technology (DGIST), Daegu, 42988, Republic of Korea
[4]Department of Mechanical Science and Engineering, University of Illinois at Urbana−Champaign, Urbana, IL 61801, USA
[5]School of Electrical and Electronic Engineering, Yonsei University, Seoul 03722, Republic of Korea
[6]Department of Bio and Brain Engineering, Korea Institute of Science and Technology (KIST), Seoul 02792, Republic of Korea

*Corresponding authors: K.-I Jang (kijang@dgist.ac.kr) and J.H.Cho (jhcho94@yonsei.ac.kr)
[†]These authors contributed equally to this work.

Supplementary Notes

Section 1. Chemical structure of the MX-CNF-PCE electrode composite

Supplementary Fig. 14 presents the simplified 3D chemical structures of MXene, cellulose nanofiber (CNF), and polycarboxylate ether (PCE). MXene, a 2D conductive material, has high electrical conductivity, but its brittleness and low oxidation stability limit its use in flexible electronics. To overcome these shortcomings, CNF was incorporated as a reinforcing agent to enhance the mechanical strength and flexibility while minimizing insulating contact between the 2D MXene nanosheets. The hydrophilic hydroxyl groups present on the CNF surfaces prevent the oxidation and moisture stability of the composites. To improve stability, MX-CNF was combined with PCE, resulting in an MX-PCE-CNF composite with enhanced resistance to oxidation and moisture. In this research, the primary electrode material, MX-CNF-PCE, comprised an MXene:CNF:PCE weight ratio of 24:4:1.

Supplementary Table 1. Multi-input CNN model details for gesture recognition.

Layer name		Kernel /Pool size	Number of filters	Stride	Padding	Output shape	Connected to
Ch1 feature extraction							
Ch1 Conv1	Conv2d	3	16	1	same	(64, 64, 16)	Ch1 Conv1
	Batch normalization/ReLU	-	-	-	-	(64, 64, 16)	
	Max Pooling	2	-	2	same	(32, 32, 16)	
Ch1 Conv2	Conv2d	3	16	1	same	(32, 32, 16)	Ch1 Conv2
	Batch normalization/ReLU	-	-	-	-	(32, 32, 16)	
	Max Pooling	2	-	2	same	(16, 16, 16)	
Ch1 Conv3	Conv2d	3	32	1	same	(16, 16, 32)	Ch1 Conv3
	Batch normalization/ReLU	-	-	-	-	(16, 16, 32)	
	Max Pooling	2	-	2	same	(8, 8, 32)	
Ch1 Flatten	Flatten	-	-	-	-	(2048)	Ch1 Flatten
Ch1 FC1	Fully connected/ ReLU/Dropout (0.3)	-	-	-	-	(256)	Ch1 FC1
Ch2 feature extraction							
Ch2 Conv1	Conv2d	3	16	1	same	(64, 64, 16)	Ch2 Conv1
	Batch normalization / ReLU	-	-	-	-	(64, 64, 16)	
	Max Pooling	2	-	2	same	(32, 32, 16)	
Ch2 Conv2	Conv2d	3	16	1	same	(32, 32, 16)	Ch2 Conv2
	Batch normalization / ReLU	-	-	-	-	(32, 32, 16)	
	Max Pooling	2	-	2	same	(16, 16, 16)	
Ch2 Conv3	Conv2d	3	32	1	same	(16, 16, 32)	Ch2 Conv3
	Batch normalization / ReLU	-	-	-	-	(16, 16, 32)	
	Max Pooling	2	-	2	same	(8, 8, 32)	
Ch2 Flatten	Flatten	-	-	-	-	(2048)	Ch2 Flatten
Ch2 FC1	Fully connected/ReLU/Dropout (0.3)	-	-	-	-	(256)	Ch2 FC1
Ch3 feature extraction							
Ch3 Conv1	Conv2d	3	16	1	same	(64, 64, 16)	Ch3 Conv1
	Batch normalization / ReLU	-	-	-	-	(64, 64, 16)	
	Max Pooling	2	-	2	same	(32, 32, 16)	
Ch3 Conv2	Conv2d	3	16	1	same	(32, 32, 16)	Ch3 Conv2
	Batch normalization / ReLU	-	-	-	-	(32, 32, 16)	
	Max Pooling	2	-	2	same	(16, 16, 16)	
Ch3 Conv3	Conv2d	3	32	1	same	(16, 16, 32)	Ch3 Conv3
	Batch normalization / ReLU	-	-	-	-	(16, 16, 32)	
	Max Pooling	2	-	2	same	(8, 8, 32)	
Ch3 Flatten	Flatten	-	-	-	-	(2048)	Ch3 Flatten
Ch3 FC1	Fully connected/ReLU/ Dropout (0.3)	-	-	-	-	(256)	Ch3 FC1
Ch4 feature extraction							
Ch4 Conv1	Conv2d	3	16	1	same	(64, 64, 16)	Ch4 Conv1
	Batch normalization/ReLU	-	-	-	-	(64, 64, 16)	
	Max Pooling	2	-	2	same	(32, 32, 16)	
Ch4 Conv2	Conv2d	3	16	1	same	(32, 32, 16)	Ch4 Conv2
	Batch normalization/ReLU	-	-	-	-	(32, 32, 16)	
	Max Pooling	2	-	2	same	(16, 16, 16)	
Ch4 Conv3	Conv2d	3	32	1	same	(16, 16, 32)	Ch4 Conv3
	Batch normalization/ReLU	-	-	-	-	(16, 16, 32)	
	Max Pooling	2	-	2	same	(8, 8, 32)	
Ch4 Flatten	Flatten	-	-	-	-	(2048)	Ch4 Flatten
Ch4 FC1	Fully connected/ReLU/Dropout (0.3)	-	-	-	-	(256)	Ch4 FC1
Ch5 feature extraction							
Ch5 Conv1	Conv2d	3	16	1	same	(64, 64, 16)	Ch5 Conv1
	Batch normalization / ReLU	-	-	-	-	(64, 64, 16)	
	Max Pooling	2	-	2	same	(32, 32, 16)	
Ch5 Conv2	Conv2d	3	16	1	same	(32, 32, 16)	Ch5 Conv2
	Batch normalization / ReLU	-	-	-	-	(32, 32, 16)	
	Max Pooling	2	-	2	same	(16, 16, 16)	
Ch5 Conv3	Conv2d	3	32	1	same	(16, 16, 32)	Ch5 Conv3
	Batch normalization / ReLU	-	-	-	-	(16, 16, 32)	
	Max Pooling	2	-	2	same	(8, 8, 32)	
Ch5 Flatten	Flatten	-	-	-	-	(2048)	Ch5 Flatten
Ch5 FC1	Fully connected/ ReLU/Dropout (0.3)	-	-	-	-	(256)	Ch5 FC1
Feature Concatenation							
Concat	Concatenated	-	-	-	-	(1280)	Concat
FC2	Fully connected/ReLU/Dropout (0.3)	-	-	-	-	(128)	FC2
FC3	Fully connected/ReLU/Dropout (0.3)	-	-	-	-	(64)	FC3
FC4	Fully connected/Softmax	-	-	-	-	(8)	FC4

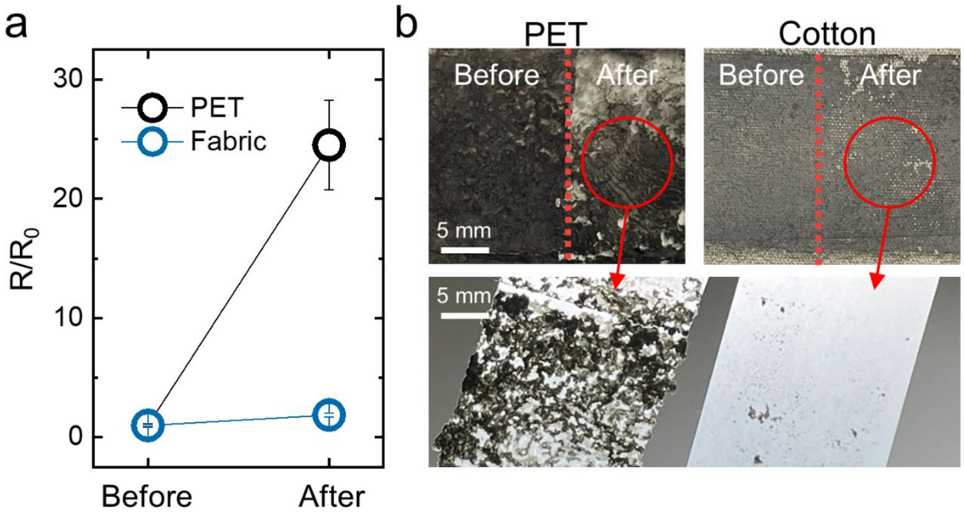

Supplementary Fig. 1. Tape test comparing PET substrate and cotton fabric with a complex weave structure. **a** Normalized resistance changes in the conductive traces of PET and cotton fabric before and after the tape test. **b** Photographs of the spray-coated PET and cotton fabric before and after the tape test.

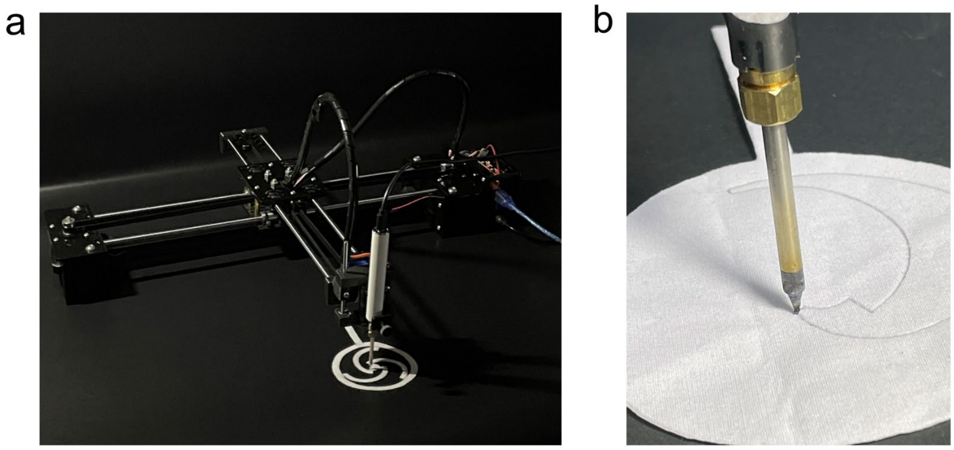

Supplementary Fig. 2. Photographs of the **a** customized XY plotter and **b** heat-sealing process with the soldering tip. Photo Credit: Sanghyun Lee, Yonsei University.

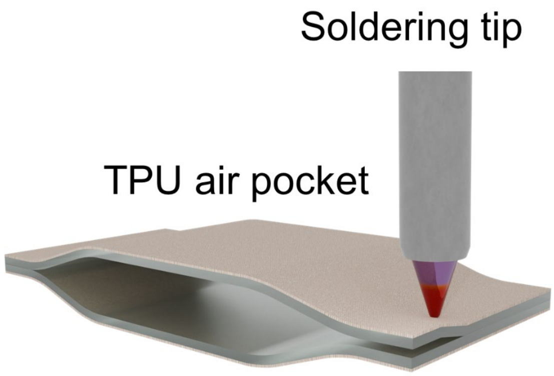

Supplementary Fig. 3. Schematic of the heat-sealing process using a soldering tip controlled by the customized XY plotter.

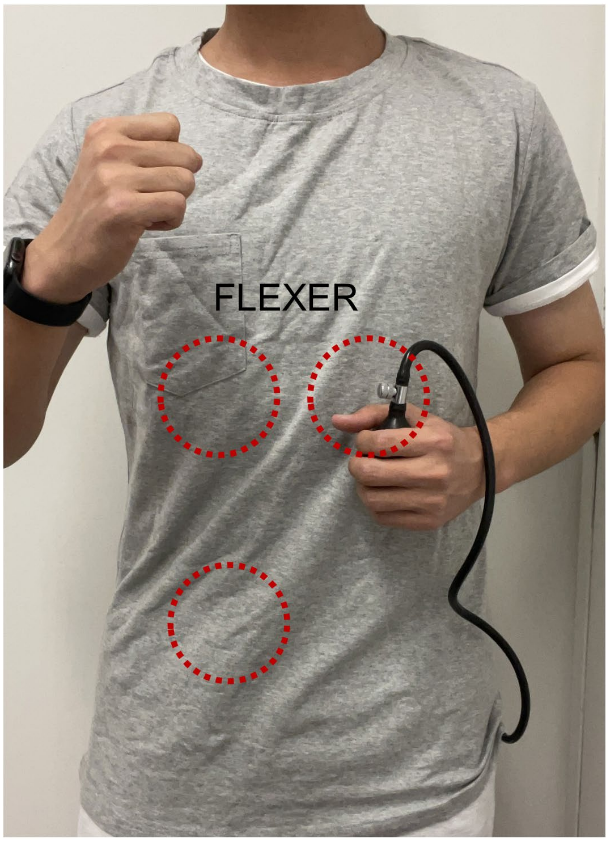

Supplementary Fig. 4. Photograph of a user wearing the ECG FLEXER-installed clothing. Photo Credit: Sanghyun Lee, Yonsei University.

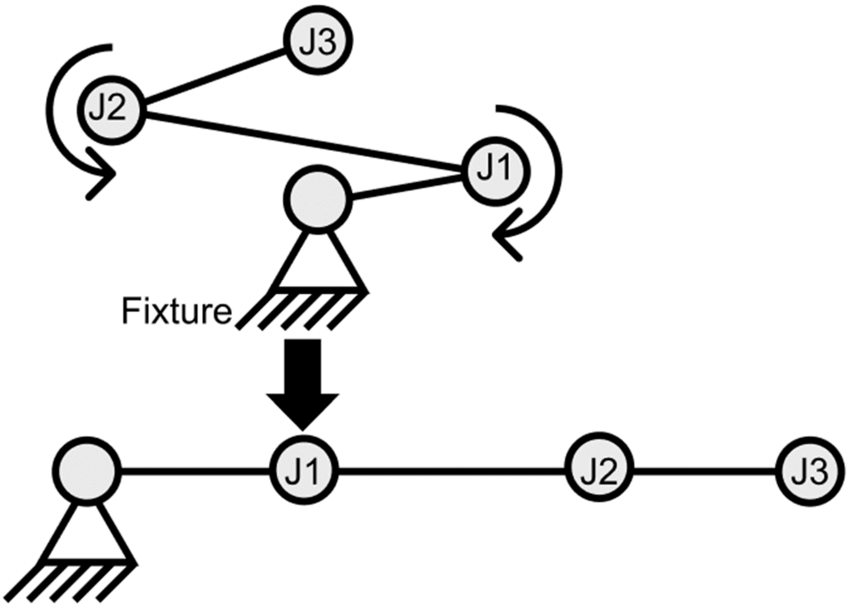

Supplementary Fig. 5. Schematic of the unfolding joint property of the FLEXER made of cotton fabric without any pneumatic force supply.

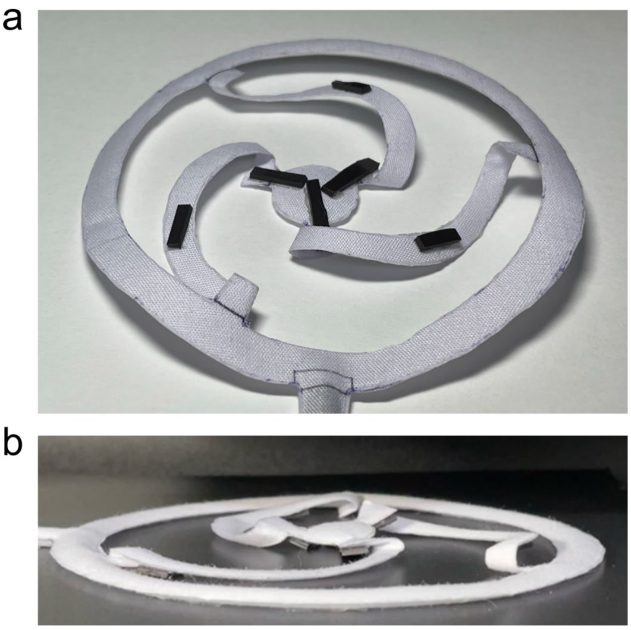

Supplementary Fig. 6. Photographs of FLEXER with rubber magnets. **a** above and **b** side view of rubber magnets attached near the joints of FLEXER to control unwanted unfolding behavior. Photo Credit: Sanghyun Lee, Yonsei University.

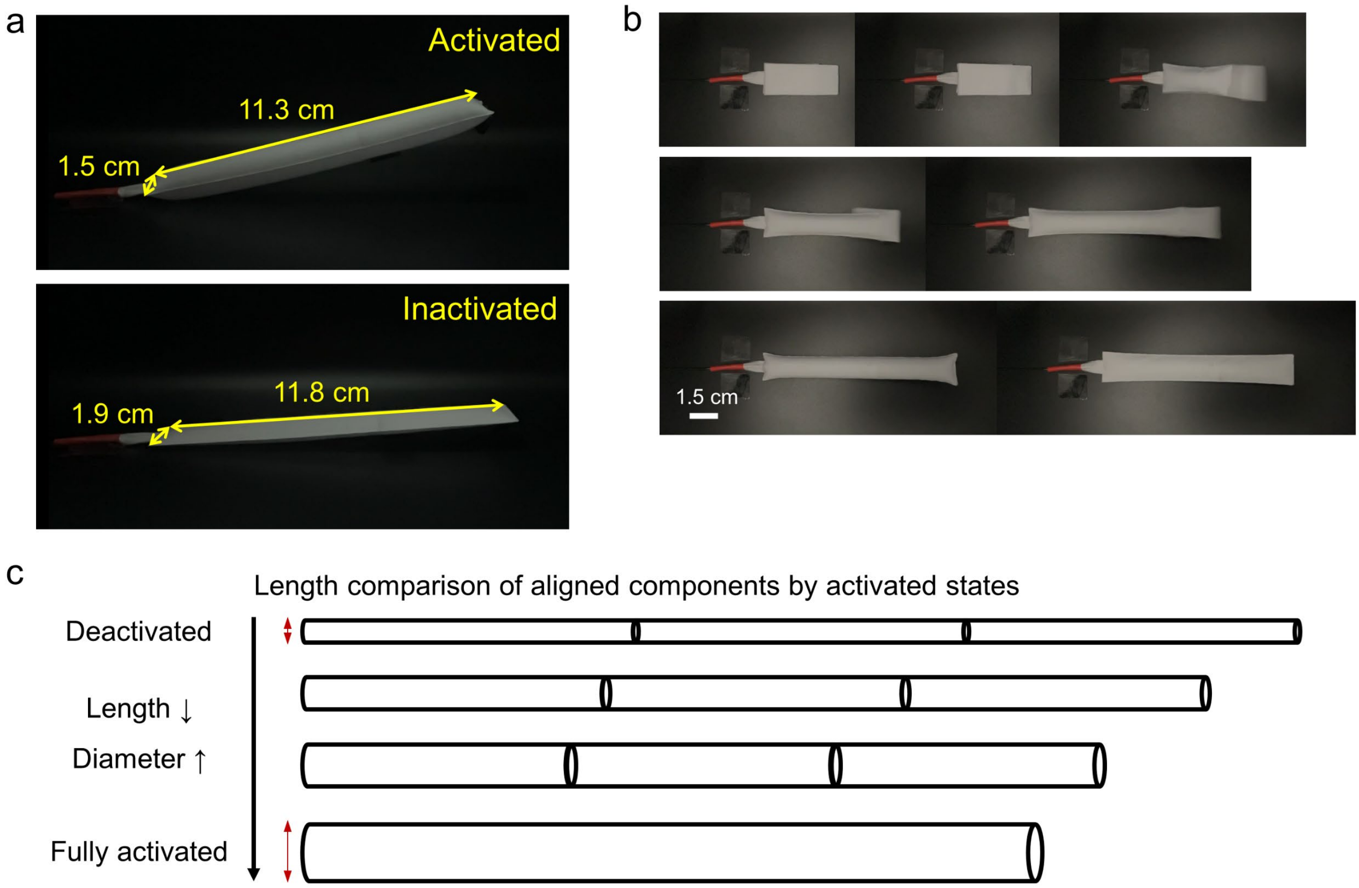

Supplementary Fig. 7. Simple Linear shape of FLEXER. Snapshots of **a** side views of activated and inactivated FLEXERs comparing their lengths and widths and **b** the full activation process. **c** Schematic explanation of the decrease in length due to perpendicular expansion induced by pneumatic force supply. Photo Credit: Sanghyun Lee, Yonsei University.

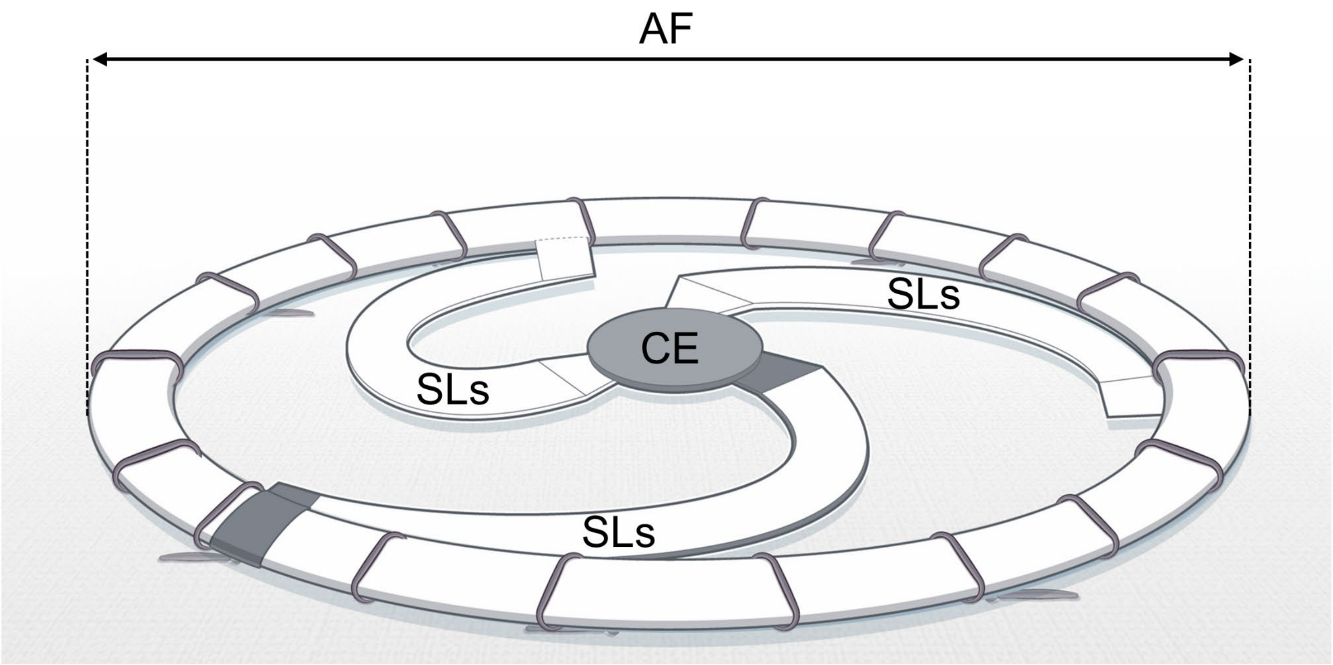

Supplementary Fig. 8. AF, SLs, and CE of FLEXER

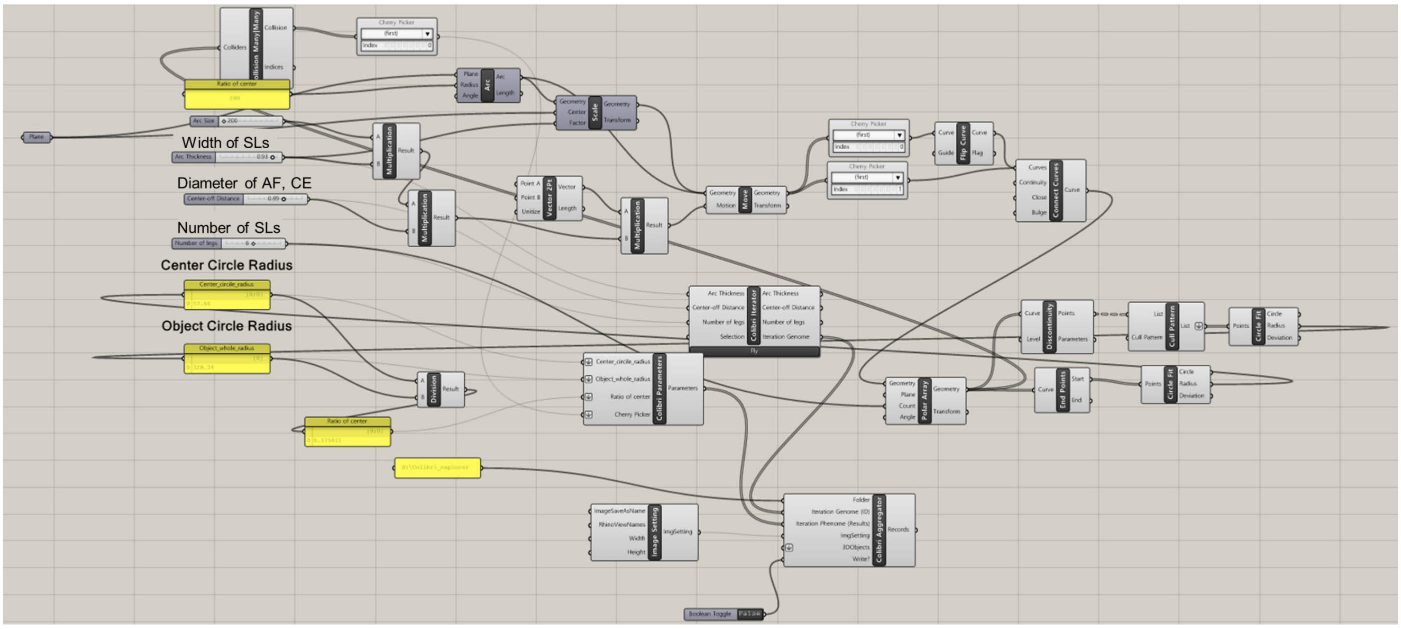

Supplementary Fig. 9. FLEXER design strategy using the visual programming software Rhino 7 and Grasshopper 3D.

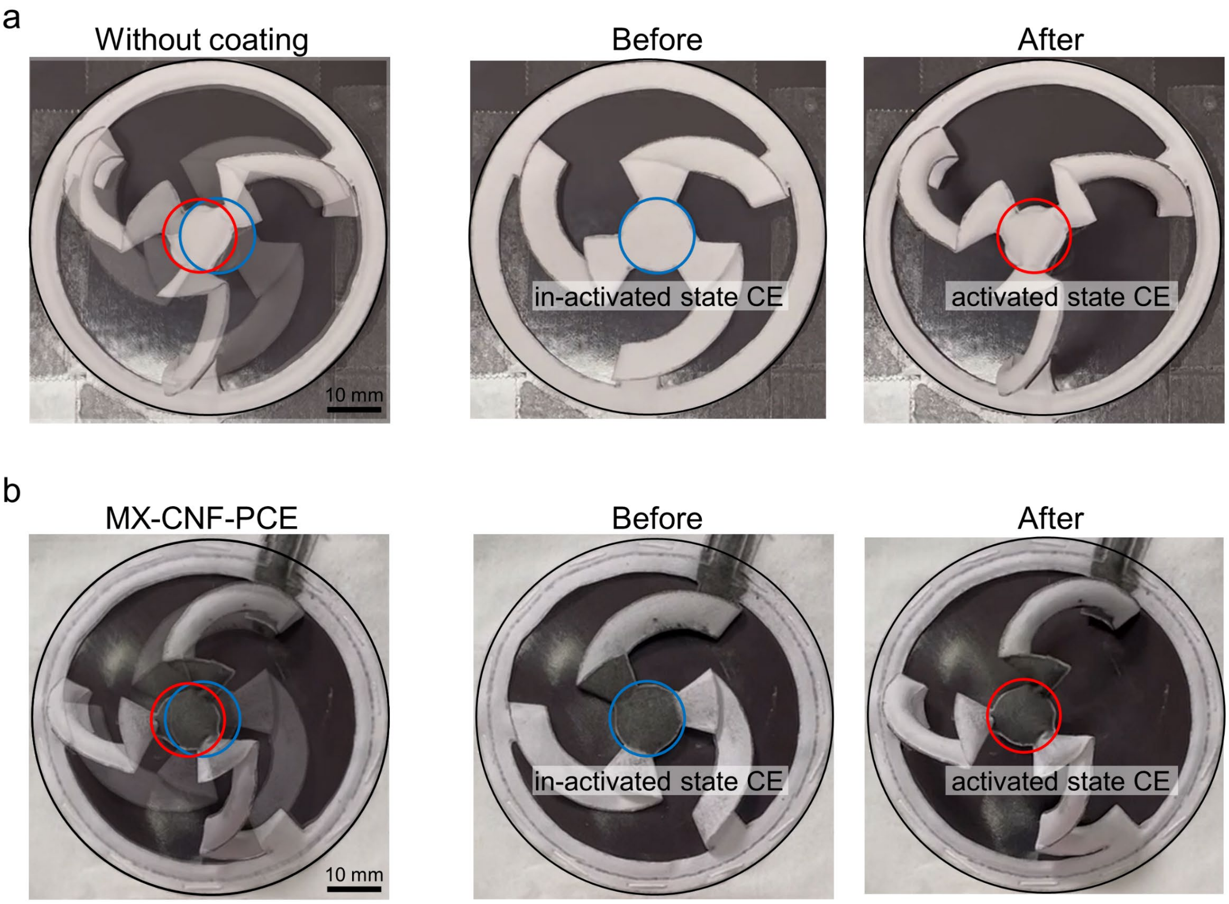

Supplementary Fig. 10. Comparison of the CE position before and after the activation of the FLEXER in cases of **a** without and **b** with an MX-CNF-PCE coating.

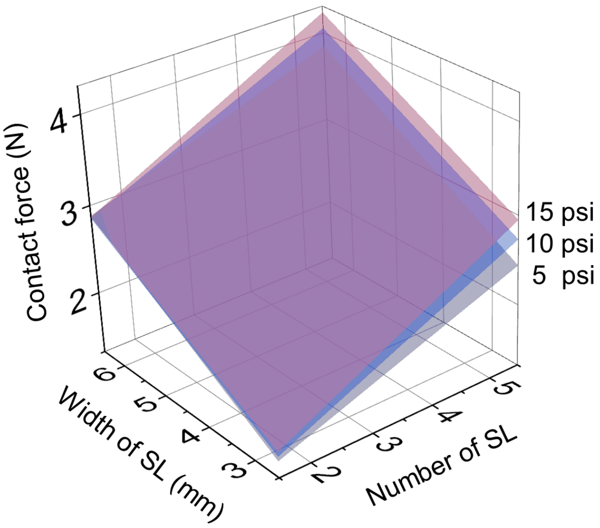

$z = 0.28+0.32x+0.34y$ (R^2=0.90, 15 psi)

$z = 0.26+0.33x+0.30y$ (R^2=0.93, 10 psi)

$z = 0.25+0.35x+0.24y$ (R^2=0.97, 5 psi)

Supplementary Fig. 11. FLEXER design equation graphs based on 5, 10, and 15 psi of pneumatic pressure. Equation of plane for each pneumatic pressure condition is written bottom the graph.

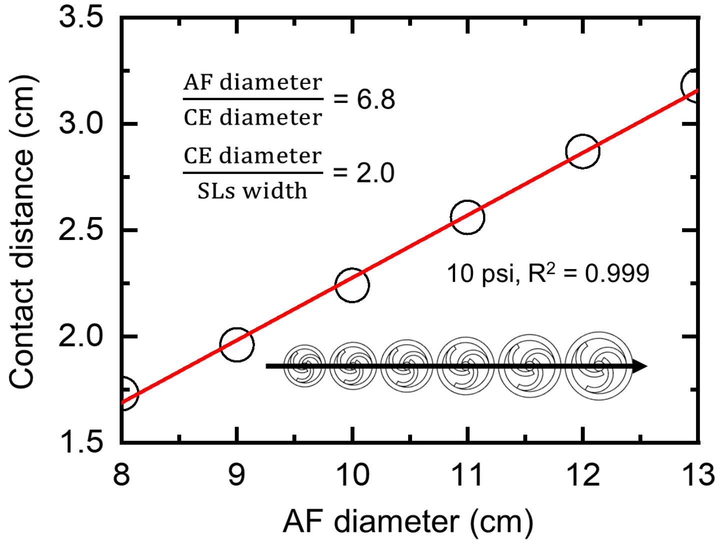

Supplementary Fig. 12. Maximum contact distances for different FLEXER sizes. The ratio of the AF diameter to the CE diameter of FLEXER is 6.8, and the ratio of the CE diameter to the SL width is 2.

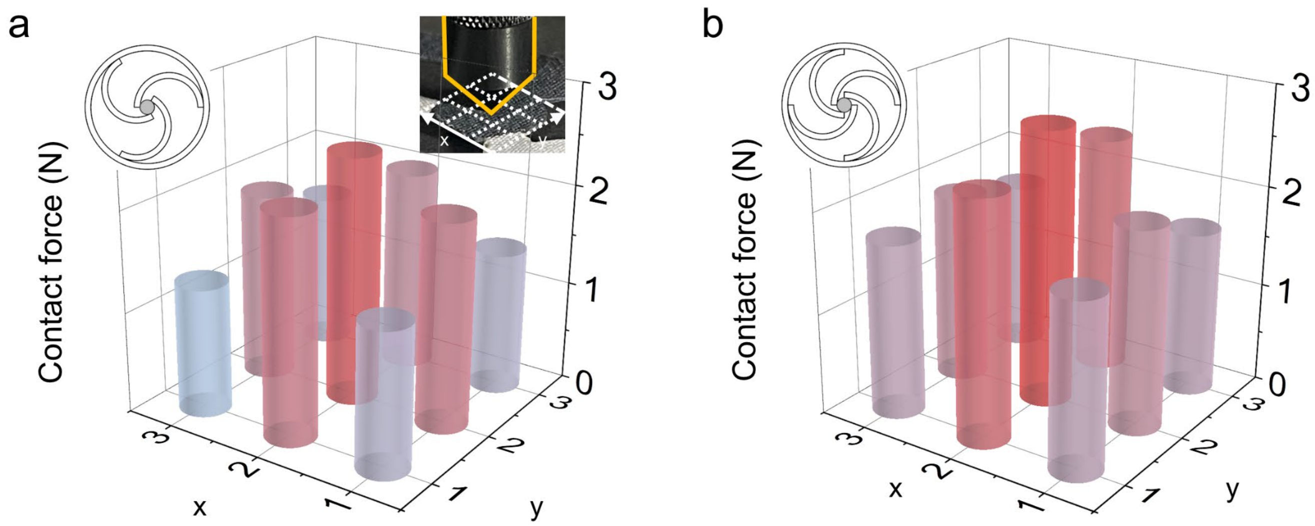

Supplementary Fig. 13. Force distributions of FLEXERs with three and four SLs. **a** Three-legged FLEXER on left with photograph of experiment setting in inset and **b** four-legged FLEXER on right.

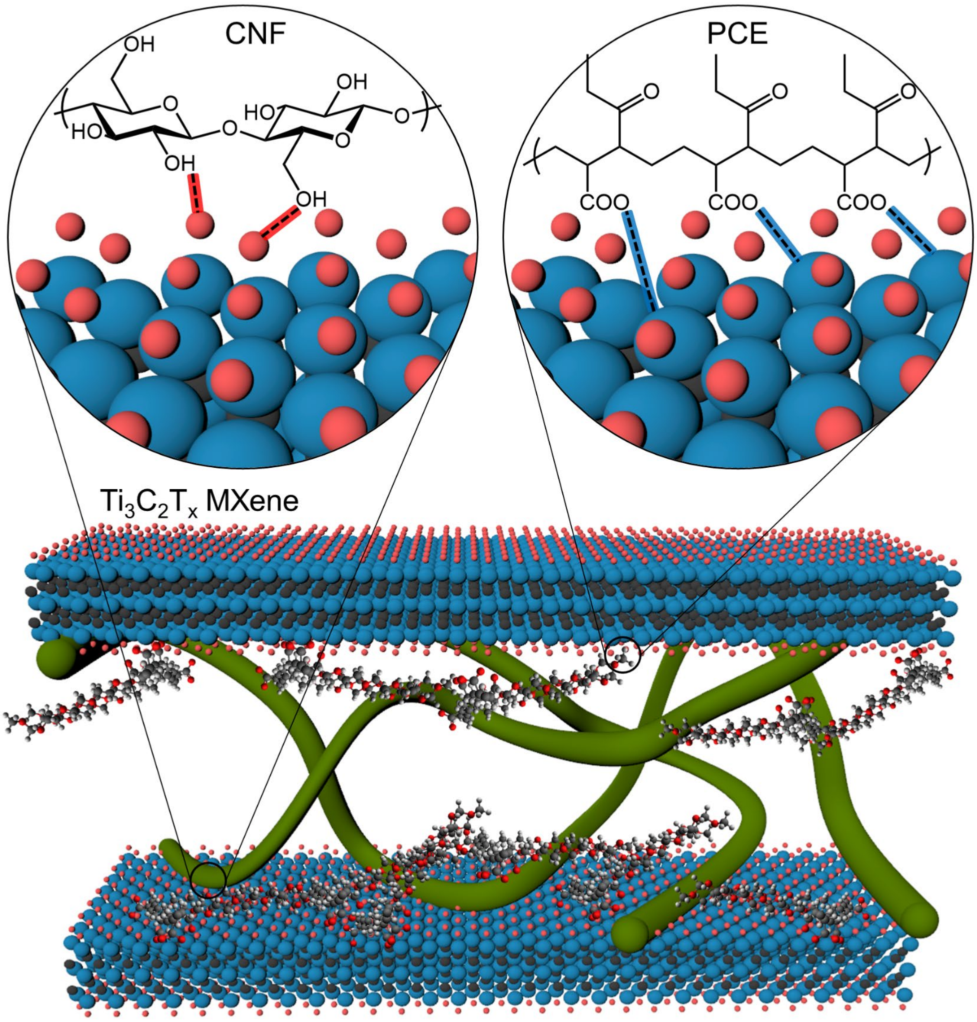

Supplementary Fig. 14. Chemical structure scheme of the MX-CNF-PCE electrode composite. MXene (diameter: several microns and thickness: 1.2–1.8 nm) has a platelet-like structure and CNF (diameter: 3–30 nm and length: several microns) serve as a platform for MXene attachment.

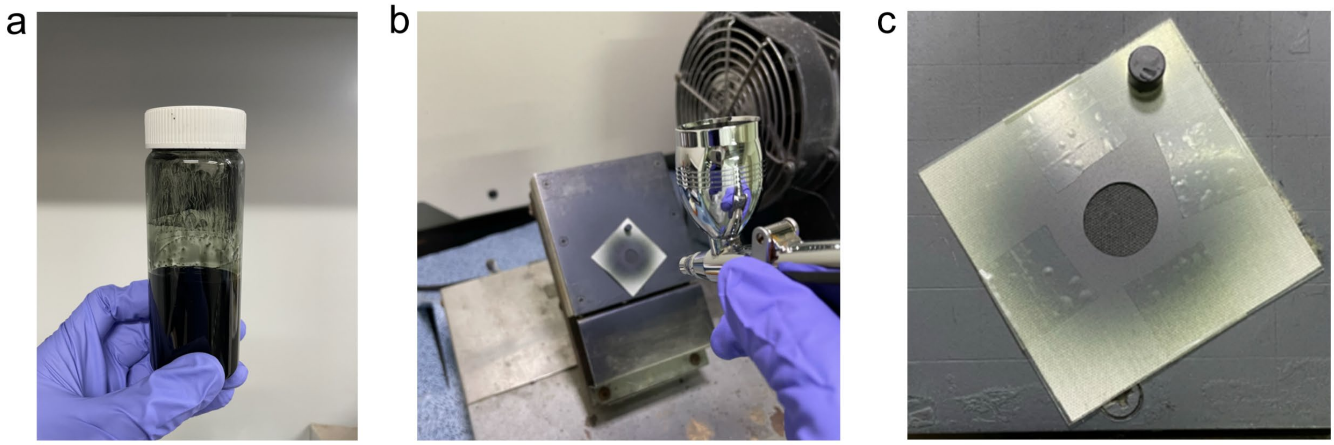

Supplementary Fig. 15. Spray-coating process for MX-CNF-PCE deposition. Photographs of the **a** MX-CNF-PCE aqueous solution, **b** MX-CNF-PCE spray-coating process on cotton fabric, and **c** MX-CNF-PCE spray-coated cotton fabric covered by a PET shadow mask. Photo Credit: Sanghyun Lee, Yonsei University.

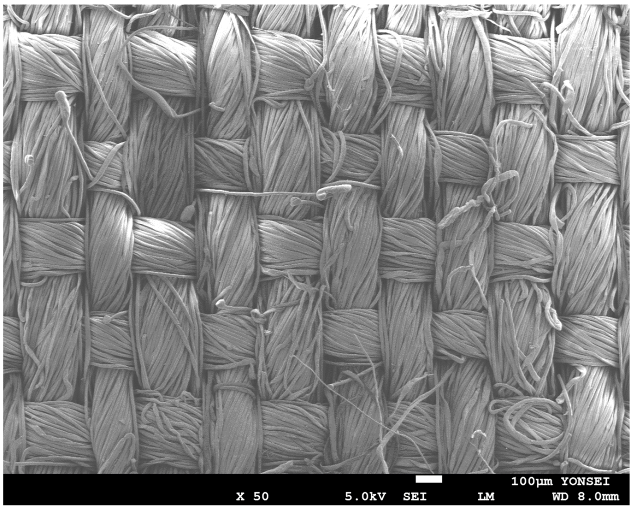

Supplementary Fig. 16. SEM image of the pristine cotton fabric.

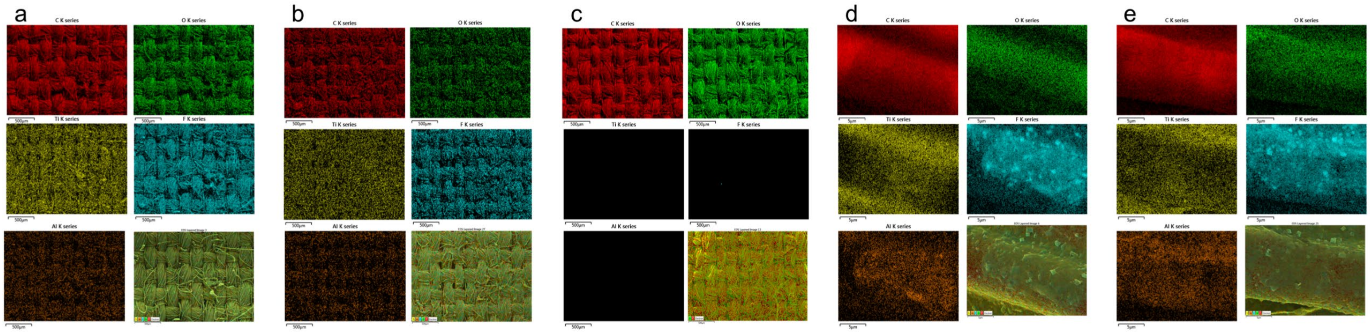

Supplementary Fig. 17. EDX mapping of 50x magnification of **a** pristine MXene, **b** MX-CNF-PCE, and **c** fabric and 5000x magnification of **d** pristine MXene and **e** MX-CNF-PCE.

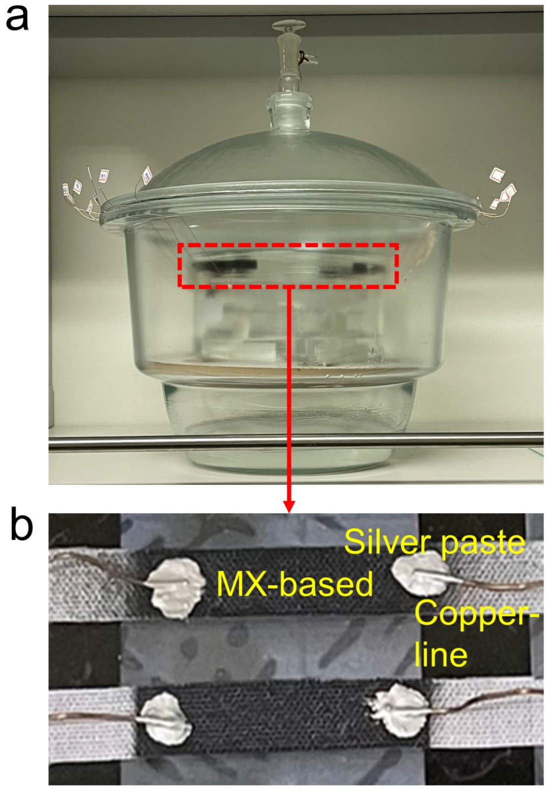

Supplementary Fig. 18. Oxidation stability test for MXene-based electrodes. Photographs of **a** a glass chamber maintaining a 100% relative humidity harsh environment and **b** spray-coated MXene-based electrodes on cotton fabric electrically connected to the outside to measure electrical resistance. Photo Credit: Sanghyun Lee, Yonsei University.

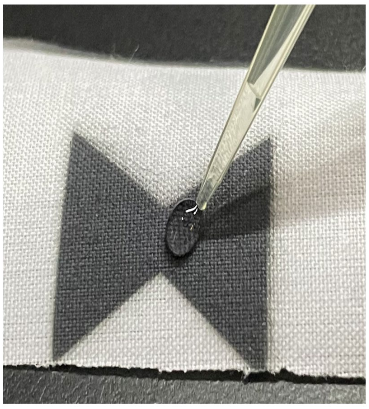

Supplementary Fig. 19. Photograph of wetting the MXene-based electrode with an artificial sweat droplet.

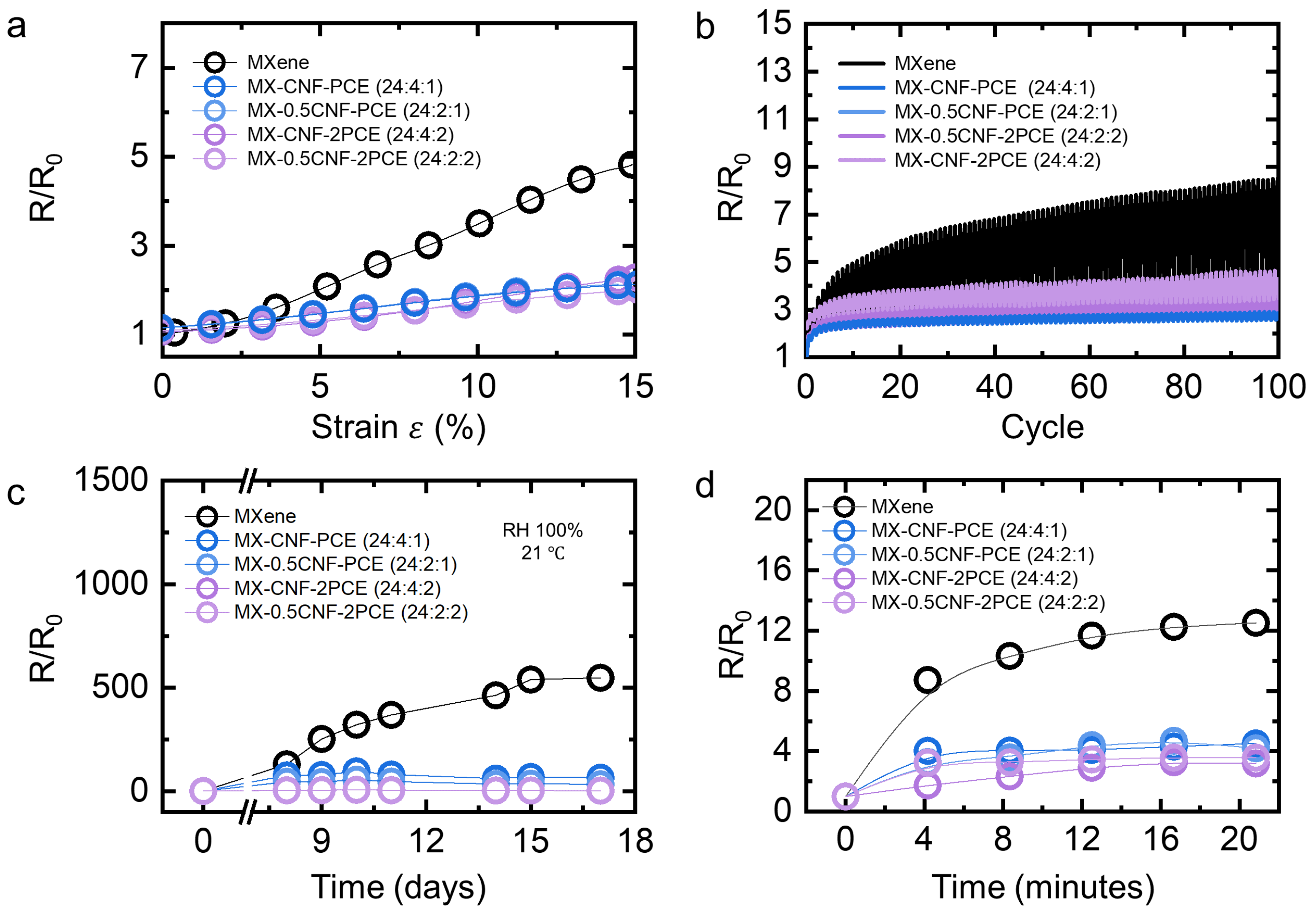

Supplementary Fig. 20. MX-CNF-PCE electromechanical property optimization test changing ratio of CNF and PCE. Normalized resistance stability measurement against **a** tensile strain, **b** cyclic bending **c** 100% humidity environment, and **d** a saline droplet.

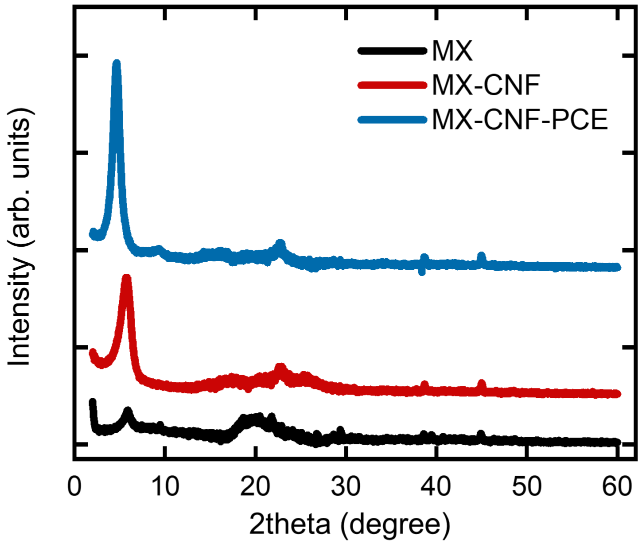

Supplementary Fig. 21. Extended XRD patterns of MX, MX-CNF, and MX-CNF-PCE.

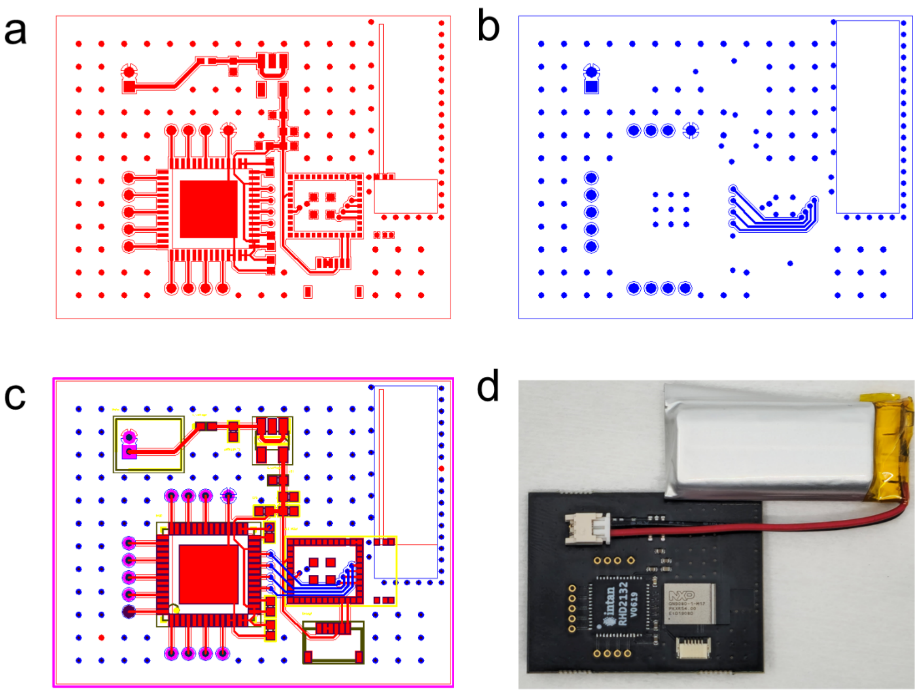

Supplementary Fig. 22. Circuit design. **a-c** Schematic illustration of the PCB artwork of the top layer (**a**), the bottom layer (**b**), and all layers (**c**). **d** Photographic images of PCB. Photo Credit: Janghwan Jekal, DGIST.

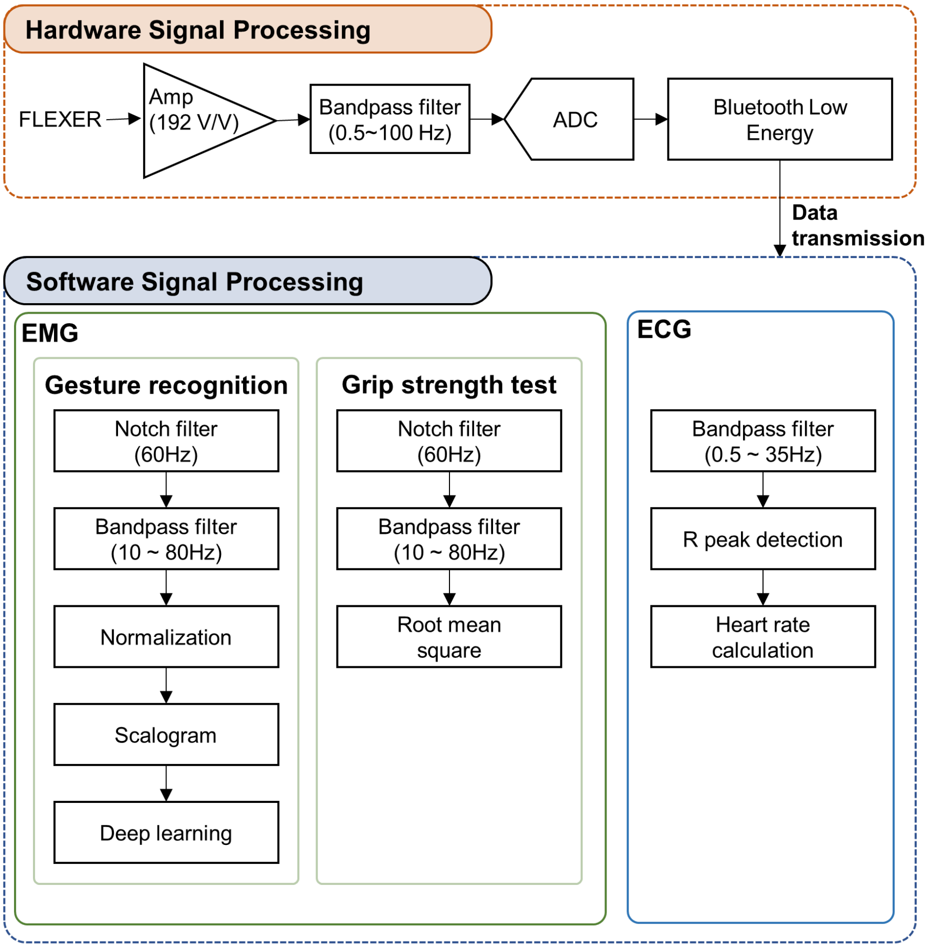

Supplementary Fig. 23. Signal processing flow of ECG and EMG signals. The ECG and EMG signals recorded by FLEXER were preprocessed through both hardware signal processing and software signal processing.

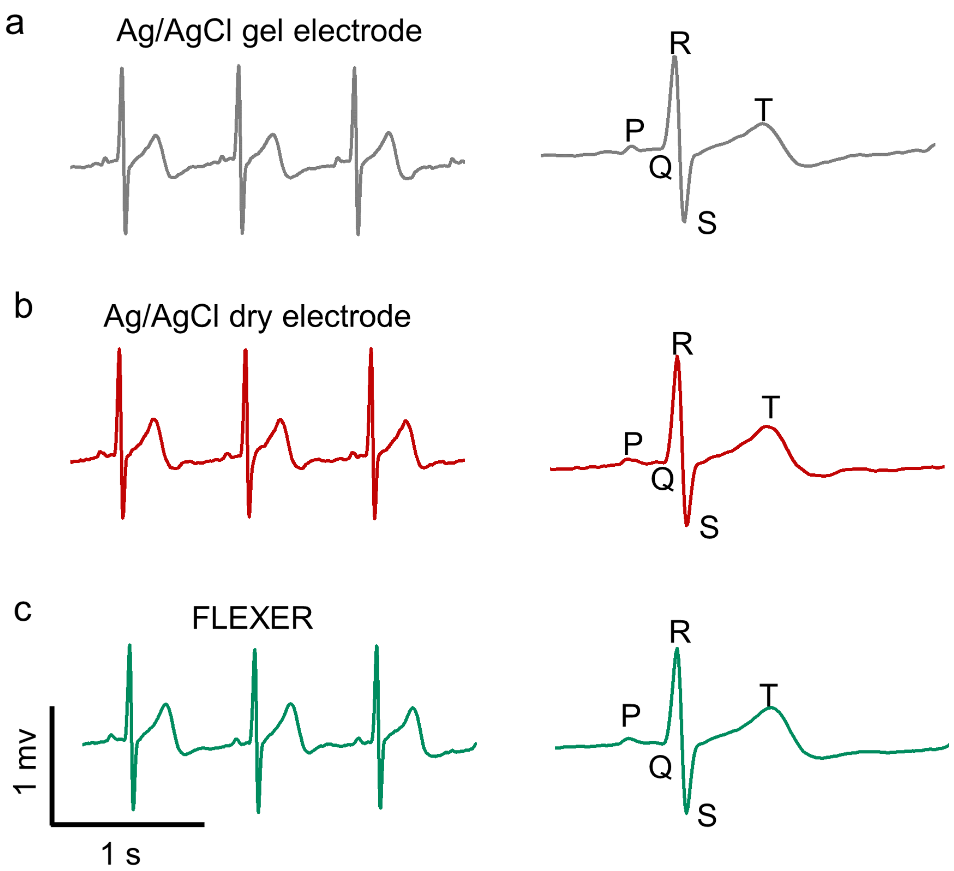

Supplementary Fig. 24. Comparison of ECG signals measured by commercial electrodes and FLEXER. **a-c** Detailed ECG signals and PQRST wave measured using the gel-type Ag/AgCl electrode **(a)**, dry Ag/AgCl electrode **(b)**, and FLEXER **(c)**.

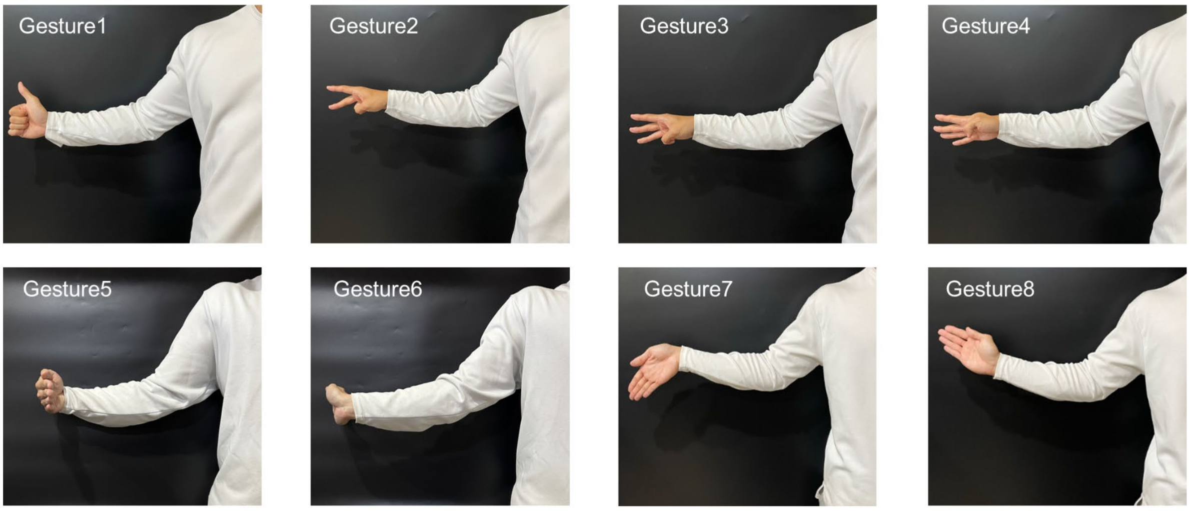

Supplementary Fig. 25. 8 types of hand gestures. Photo Credit: Sanghyun Lee, Yonsei University.

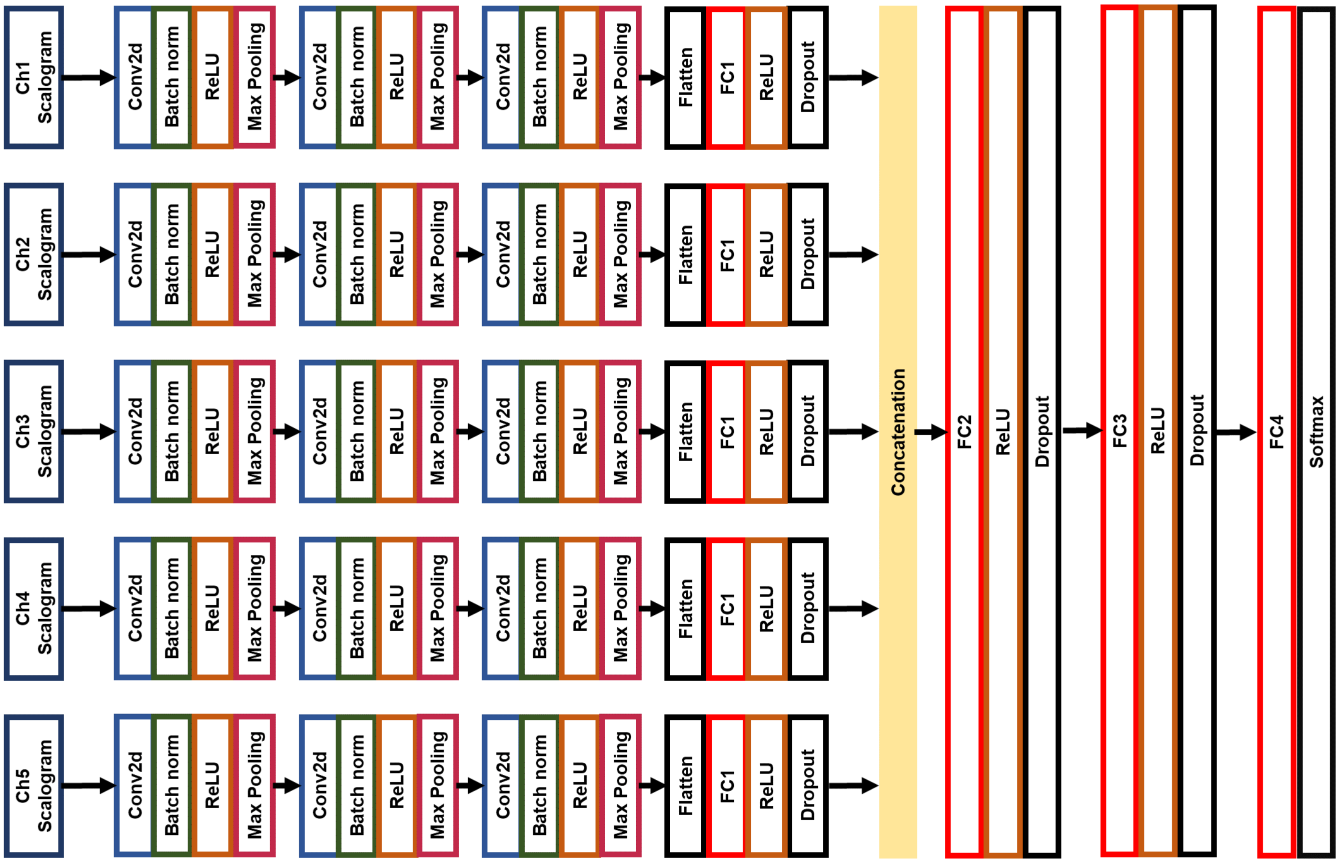

Supplementary Fig. 26. Layer architecture of the multi-input neural network model. The architecture of the concatenated neural network for hand gesture recognition.

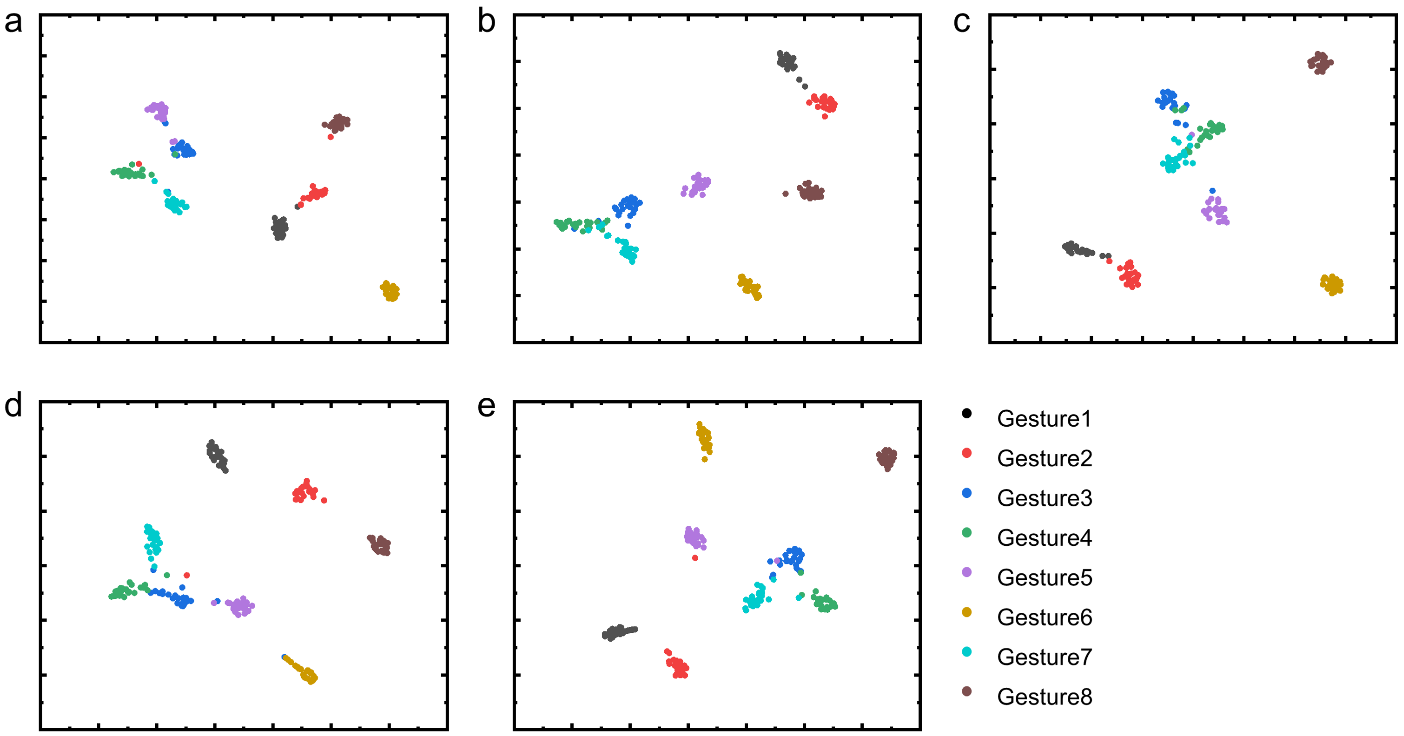

Supplementary Fig. 27. Detailed visualization of the t-SNE results. Each gesture is depicted by uniquely colored points. **a** Fold 1, **b** Fold 2, **c** Fold 3, **d** Fold 4, and **e** Fold 5.

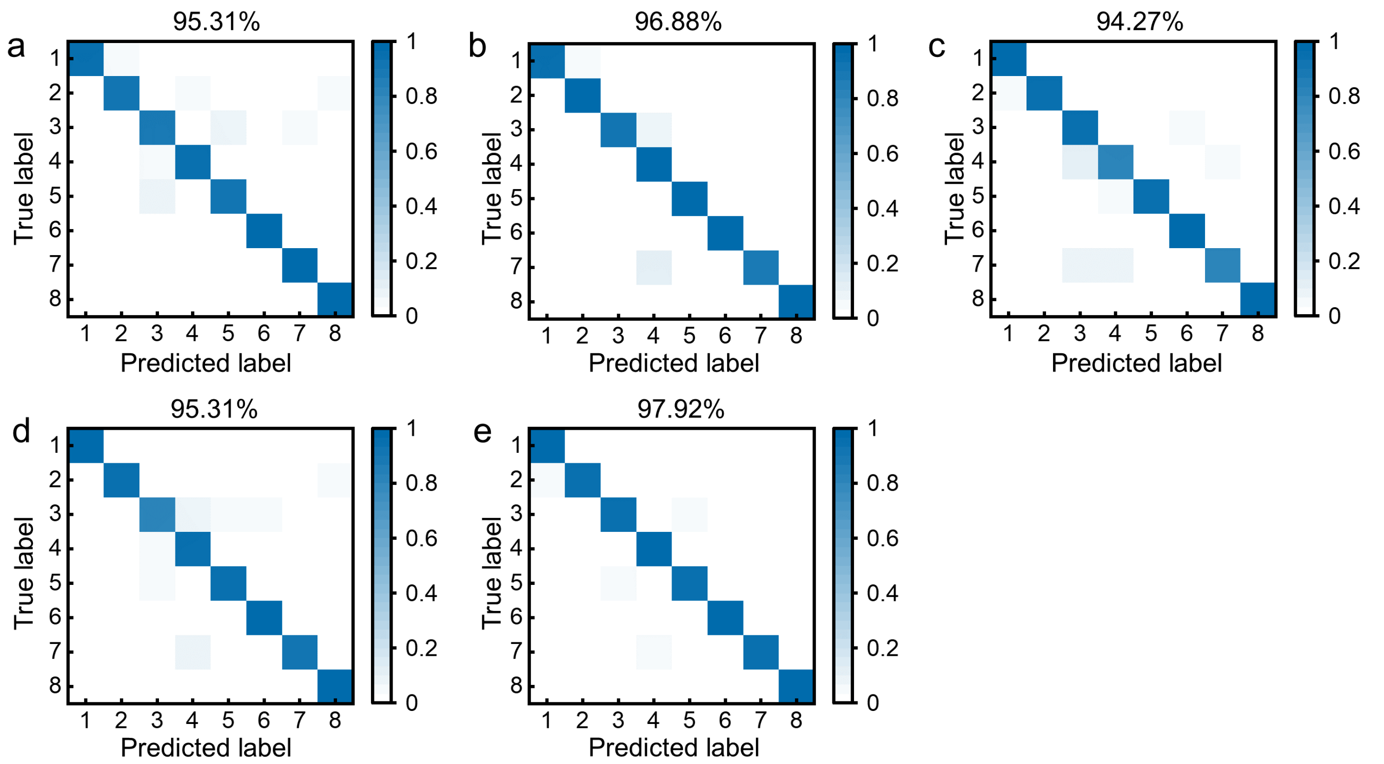

Supplementary Fig. 28. Confusion matrices of the gesture recognition results across all folds with accuracy. **a** Fold 1, **b** Fold 2, **c** Fold 3, **d** Fold 4, and **e** Fold 5.

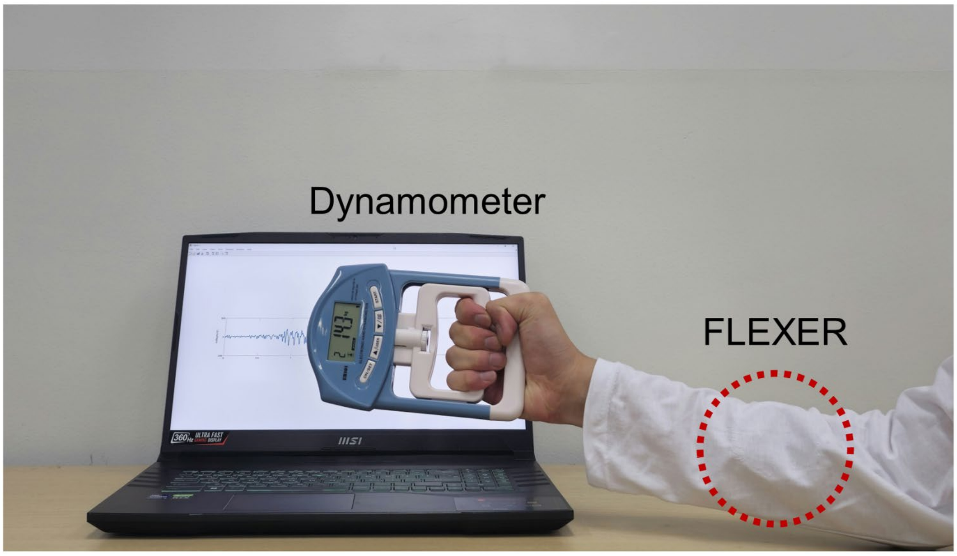

Supplementary Fig. 29. Grip strength test. Photo Credit: Janghwan Jekal, DGIST
