# Supplementary material for: Fabric-based lamina emergent MXene-based electrode for electrophysiological monitoring
Source: Nat Commun. 2024 Oct 2;15:5974. doi: 10.1038/s41467-024-49939-x (PMC11446925; doi:10.1038/s41467-024-49939-x)
Supplement: Supplementary file 3 — Description of Additional Supplementary Files [file 41467_2024_49939_MOESM3_ESM.pdf]

Description of Additional Supplementary Files

Supplementary Movie 1. Video analysis of linear PIA actuation

Supplementary Movie 2. Actuation and collapse of linear PIA actuator

Supplementary Movie 3. Contact stability test of balloon

Supplementary Movie 4. Contact stability test of FLEXER

Supplementary Movie 5. Single & Multi-FLEXER inflation demonstration

Supplementary Movie 6. Real-life ECG monitoring
